# Knowledge of pharmacy students about doping, and the need for doping education: a questionnaire survey

**DOI:** 10.1186/s13104-017-2713-7

**Published:** 2017-08-11

**Authors:** Keita Shibata, Koichi Ichikawa, Naomi Kurata

**Affiliations:** 1 Division of Community Healthcare and Pharmacy, Department of Healthcare and Regulatory Sciences, School of Pharmacy, Showa University, 1-5-8 Hatanodai, Shinagawa-ku, Tokyo, 142-8555 Japan; 2 Division of Pharmacology, Department of Pharmacology, Toxicology and Therapeutics, School of Pharmacy, Showa University, Tokyo, Japan

**Keywords:** Pharmacy students recognition, Sports pharmacist, Doping, Education

## Abstract

**Background:**

Anti-doping activities are carried out on a global scale. Based on these activities, the specialty of “sports pharmacist,” which entails a deeper comprehension of doping, use of supplements, and appropriate drug use for athletes, was established in 2009 in Japan. It is difficult to say whether the education on doping is adequate for pharmacy students who will be eligible to become sports pharmacists. It is also unclear how well these students understand doping. Therefore, the aim of this study was to investigate pharmacy students’ current knowledge of appropriate drug use, doping and use of supplements, and to explore the need for further education on these topics.

**Methods:**

A questionnaire survey was conducted from July 3rd to August 2nd in 2014 at Showa University in Japan. A total of 406 respondents (2nd- to 6th-year students) were assessed as eligible. Group comparison was used to compare those who had attended a lecture about doping and those who had not.

**Results:**

Most of the students only knew the word doping and had not attended a lecture on the subject, but 72% of them expressed a desire to attend one. Over half did not know that the most common doping violation in Japan is unintentional doping, and were unfamiliar with certain past cases of doping. In addition, 41% did not know that over-the-counter medicines and dietary supplements might contain prohibited substances, and 87% were unaware that names of prohibited substances might not appear on the ingredient labels of dietary supplements. In contrast, attending a lecture on doping was effective in facilitating the acquisition of all these types of knowledge.

**Conclusions:**

It is important to provide more opportunities for appropriate education of pharmacy students on the topic of doping, given that interest exists and attending a lecture on the topic appears to be useful. More education about doping for pharmacy students would be as effective for anti-doping activities as is education of athletes.

## Background

For athletes across a wide spectrum of sports, the use and abuse of medicines, dietary supplements and other substances has become a common and problematic phenomenon. Doping is not only the use or attempted use of prohibited substances or methods by athletes, but also can be committed by anyone who assists in, encourages, aids, abets, conspires with, or covers up a doping rule violation, or engages in any type of international complicity involving an anti-doping rule violation or any attempted violation. Such activity is strictly prohibited by the World Anti-Doping Code (WADC) [1]. However, anti-doping rule violation shows no sign of significant decrease in recent athletic events, including the Olympic Games [2–4].

Because of expansion of the eligibility rules for doping tests, such tests are now conducted not only at top-level international competitions but also at domestic-level events regardless of participants’ ages. It may be a particularly significant social problem among younger generations [5]. According to the WADC, a substance or method should be considered for inclusion on the prohibited list if the World Anti-Doping Agency (WADA), which has sole discretion in the matter, determines that the substance or method meets any two of the following three criteria: (1) potentially enhance or actually enhances sport performance, (2) represents an actual or potential health risk to athletes, (3) violates the spirit of sport [1]. It is also important that not only athletes but also the public have knowledge regarding both appropriate use of substances and anti-doping activities, because this knowledge could lead to a better understanding of substance abuse. However, there is little information on doping in general, and public’s access to such information is also limited.

In the case of unintentional doping, which accounts for most anti-doping violations [6, 7], athletes take prohibited substances because of a lack of knowledge regarding doping and regulations. In Japan, there are many easily accessed over-the-counter (OTC) medicines that contain prohibited substances. Although all athletes are expected to recognize and understand the WADC regulations, this is sometimes very difficult; therefore, the help of more knowledgeable professionals, such as pharmacists, is needed.

The Japan Anti-Doping Agency (JADA) approved the “Sports Pharmacist Accreditation Program” in 2009 in a bid to produce pharmacists who have deeper comprehension of doping, use of supplements, and appropriate drug use for athletes. The sports pharmacist system is to certify the qualified pharmacists who are trained to have accurate knowledge in anti-doping and who can provide the appropriate information on medicine and the effects of drugs on health [8]. It is a fully-qualified and licensed pharmacist, and no age restriction. The applicants should complete both “basic” and “practical” courses delivered by JADA and obtain the required minimum score in the exam. The qualified sports pharmacist must annually take part in a practical lecture course in order to maintain a certification. Therefore, pharmacists who are specialists in drug use for the public must participate in anti-doping activities as a “sports pharmacists” [7]. The sports pharmacist has a pivotal role in promoting the prevention of doping at its roots. Thus, anti-doping activities in Japan were implemented as a government-led collaboration to keep pace with progress being made in this area throughout the world. On the other hand, sports pharmacists are approved by JADA, but they do not have jobs they can only do with that, but they are useful when they work at a community pharmacy.

On the other hand, in response to Japanese social needs, the 6-year pharmacy education system, completion of which is a prerequisite for registration for the pharmacy licensing examination, was established under the Pharmacists Law and the Fundamentals of Education Act in the 2006 academic year. All Japanese Universities that have a school of pharmacy initiated the new education system based on a model core curriculum for the pharmacy education and pharmacy practice programs [9, 10]. In 2013, to accommodate other developments in the medical environment, that model core curriculum was revised. A new education system based on this revision was implemented in 2015 [11]. It was also required that each University have unique subjects relating to pharmaceutics. Based on the establishment of a sports pharmacist system, some Universities may choose to prepare a curriculum that addresses the relationship of sports to medicine, which could include doping. Athletes are not only the top athletes but also people including children and amateur players. The latter athletes also need to know about doping for their health and avoid unintentional doping for the future. In addition, they consult with a pharmacist in community pharmacy on a daily basis to assist in the selection of medication and might purchase medicines there. Therefore, because all pharmacists might have an opportunity to advise them at general community pharmacy, an educational program in which pharmacy students can learn about the risk of doping and the use of supplements is important for training effective pharmacists in the future [12].

Therefore, the aim of the present study was to investigate the current knowledge and awareness of appropriate drug usage, including doping and use of supplements, among pharmacy students in Japan, and to explore the need for education in this area.

## Methods

### Ethical considerations

This study was approved by the Ethics Committee of the Showa University School of Pharmacy (No. 198) and was conducted in accordance with Japanese ethical guidelines for epidemiological research and the Declaration of Helsinki. All data were collected after obtaining informed verbal consent from respondents.

### Study design

A questionnaire survey was conducted at Showa University in Japan from July 3rd to August 2nd in 2014. The survey was carried out in the form of a questionnaire comprising five sections to elucidate pharmacy students’ recognition, knowledge and willingness about doping. The first section was about the general recognition of doping; the second one was about knowledge of any past doping cases; the third was about the prohibited substances included in OTC medicines and dietary supplements; the fourth involved the relationship between athletes and the medical professions regarding anti-doping activities; the fifth dealt with willingness to attend a lecture about doping in the future. A sample of 830 students was employed voluntarily from all 1012 2nd- to 6th-year students at Showa University. Their identifying information was not taken, and students indicated their consent by participating in this study without their signature after reading a document that described the study. Table 1 lists the survey items. They chose it with Yes or No for Q1–6, 8, 9 and 12, or chose it among options for Q7, 10 and 11. We excluded 424 respondents who did not answer till the end of the questionnaire and answered only a few questions. Because they stopped responding on the way and it could not be used enough for analysis, we excluded them from the present study. Data from the 406 remaining respondents were deemed eligible for analysis.

**Table 1:** Survey questions

Q1. Do you know the word “doping”?
Q2. Have you ever attended a lecture about doping before?
Q3. Do you think that athletes are permitted to use the prohibited substance to improve their performance?
Q4. Do you think athletes were never permitted to use the prohibited substance for medical treatment?
Q5. Did you know that the most common doping violation in Japan is unintentional doping?
Q6. Do you know of any past cases of doping?
Q7. What was the past case of doping that you know of? e.g. “intentional doping” or “unintentional doping”. e.g. “international competition” or “domestic competition”
Q8. Did you know that OTC medicines and dietary supplements might contain prohibited substances?
Q9. Did you know that the names of prohibited substance might not appear on the ingredients label on dietary supplements?
Q10. Who do you think should mainly get involved in anti-doping activities for athletes? e.g. “Doctor”, “Dentist”, “Pharmacist”, “Nurse”, Physical therapist”, Occupational therapist”, Nutritionist”, “Coach”, “Trainer”, or “others”
Q11. Who do you think should be mainly as an advisor about dietary supplements? e.g. “Doctor”, “Dentist”, “Pharmacist”, “Nurse”, Physical therapist”, Occupational therapist”, Nutritionist”, “Coach”, “Trainer”, or “others”
Q12. Do you want to attend a lecture about doping in the future?

### Statistical analysis

All statistical analysis was performed with IBM SPSS v.22 (Tokyo, Japan). Group comparison was used to compare those who had attended a lecture about doping with those who had not. Statistical significance was evaluated using Fisher’s exact test, with *p* < 0.05 considered statistically significant.

## Results

### Characteristics of respondents

In total, 81 respondents (21%) had attended a lecture about doping (Previous Lecture group, PL); 305 (79%) had not (No Previous Lecture Group, NPL). Table 2 shows the distribution of sex and grade of respondents in each of these two groups. Pharmacy school in Japan is experiencing a gender imbalance at other University as well, which means the number of women is higher than that of men [13].

**Table 2:** Characteristics of eligible respondents

	Total	Know about doping	Have attended a lecture		Have not attended a lecture	
	n	n	n	%	n	%
Sex						
Male	84	81	19	23	62	77
Female	322	305	62	20	243	80
Total	406	386	81	21	305	79
Grade						
2nd year	113	101	6	6	95	94
3rd year	79	79	1	1	78	99
4th year	65	60	10	17	50	83
5th year	71	71	35	49	36	51
6th year	78	75	29	39	46	61
Total	406	386	81	21	305	79

### General recognition of doping

**Fig. 1:**
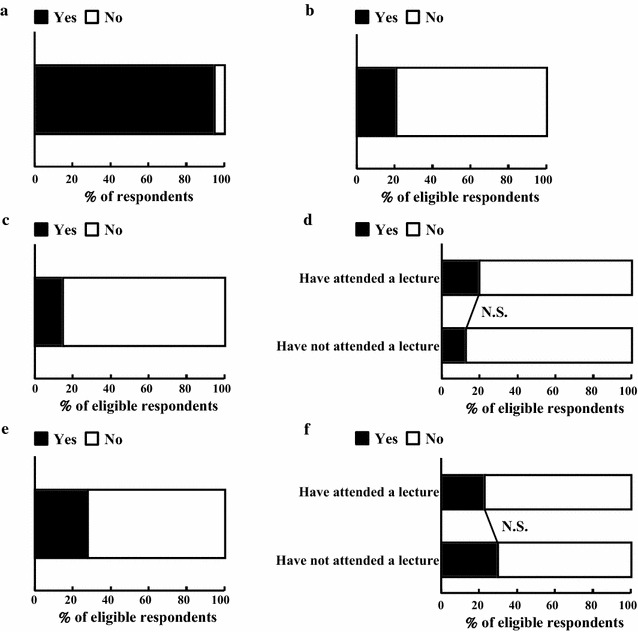
Results for general recognition of doping facts. **a** Q1 (n = 406). **b** Q2 (n = 386). **c** Q3 (n = 386). **d** Q3 group comparison comparing those who had (n = 81) and those who had not (n = 305) attended a lecture about doping. **e** Q4 (n = 386). **f** Q4 group comparison comparing those who had (n = 81) and had not (n = 305) attended a lecture about doping

Results regarding the general recognition of doping (Table 1, Q1–4) are shown in Fig. 1. First, recognition of the word of doping was confirmed (Table 1, Q1): 386 respondents (95%) said that they were familiar with the word (Fig. 1a). Of those who answered yes to Q1, 81 (21%) were in the PL group (Table 1, Q2; Fig. 1b). Group comparison was conducted based on this result.

Respondents were asked about athletes’ taking prohibited substances or medications (Table 1, Q3 and Q4). Fifty-seven respondents (15%) thought that athletes might choose to use prohibited substances to improve their performance (Fig. 1c). Of these, 16 (20%) were in the PL group and 41 (13%) in the NPL group (Fig. 1d). This group difference was not significant. In contrast, 110 respondents (28%) held the view that athletes were never permitted to use prohibited substances, even for medical treatment (Fig. 1e). Of these, 19 (23%) were in the PL group and 91 (30%) in the NPL group (Fig. 1f). Again, the difference was not significant.

### The recognition of past cases of doping

**Fig. 2:**
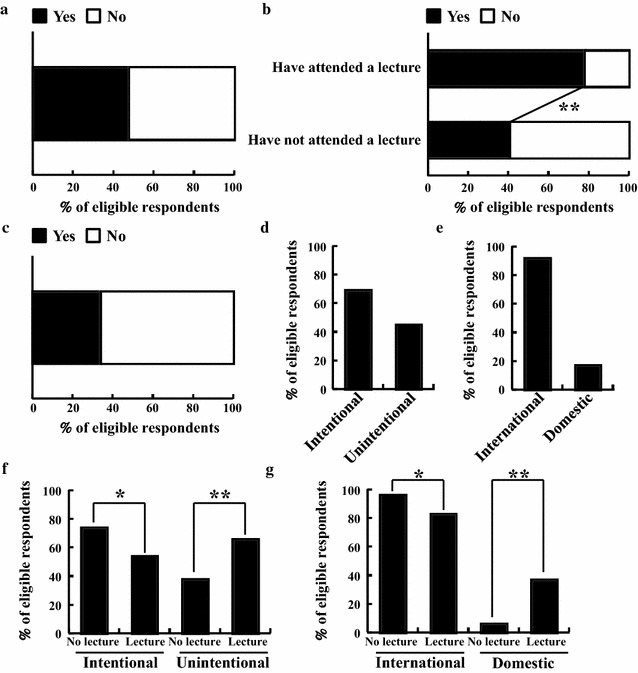
Results for recognition of past doping cases. **a** Q5 (n = 386). **b** Q5 group comparison comparing those who had (n = 81) and had not (n = 305) attended a lecture about doping. **c** Q6 (n = 386). **d**, **e** Q7 (n = 133). **f**, **g** Q7 group comparison comparing those who had (n = 35) and had not (n = 98) attended a lecture about doping. *p < 0.05 and **p < 0.01, statistically significant group differences

Results regarding the recognition of past doping cases (Table 1, Q5–7) are shown in Fig. 2. Nearly half of respondents (187, 48%) correctly recognized that the most common doping violation in Japan is unintentional doping (Table 1, Q5; Fig. 2a). Of these, 63 (78%) were PL and 124 (41%) were NPL (Fig. 2b). This group difference was significant (*p* < 0.01). About a third of respondents (133, 34%) said that they knew about past cases of doping (Table 1, Q6; Fig. 2c). Among those who answered yes to Q6, 92 (69%) were PL; 60 (45%) were NPL (Table 1, Q7; Fig. 2d). When asked about their knowledge of doping cases at competitions, 123 (92%) knew about such cases at international competitions, and 22 (17%) knew of cases at domestic competitions (Table 1, Q7; Fig. 2e). Group comparison using these results was conducted. The number of PL respondents with knowledge of cases of intentional doping was 19 (54%); for the NPL group it was 73 (74%) (Fig. 2f). This group difference was significant (*p* < 0.05). Similarly, the number of PL group members who knew of past cases of unintentional doping was 23 (66%); of the NPL group, 37 (38%) knew of such cases (Fig. 2f). Again, this difference in favor of the NPL group was statistically significant (*p* < 0.01). For doping cases at international competitions, 29 (83%) of the PL group and 94 (96%) of the NPL group reported knowing about such cases (Fig. 2g); this difference was also significant (*p* < 0.05). For doping cases at domestic competitions, the PL group showed a higher level of knowledge than did the NPL group: 13 (37%) vs. 6 (6%), respectively (*p* < 0.01) (Fig. 2g).

### Prohibited substances included in OTC medicines and dietary supplements

**Fig. 3:**
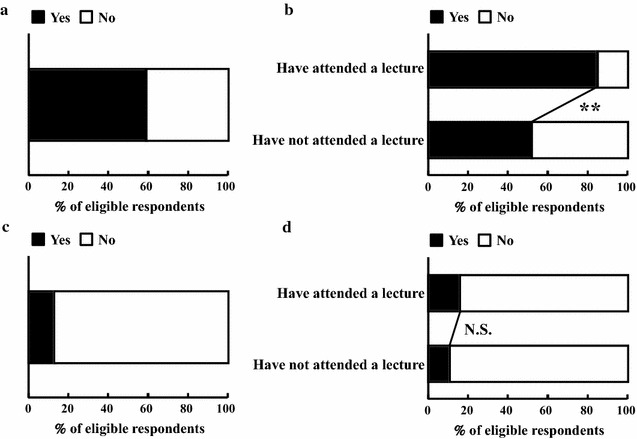
Results related to knowledge of prohibited substances included in OTC medicines and dietary supplements. **a** Q8 (n = 386). **b** Q8 group comparison comparing those who had (n = 81) and had not (n = 305) attended a lecture about doping. **c** Q9 (n = 228). **d** Q9 group comparison comparing those who had (n = 69) and had not (n = 159) attended a lecture about doping. **p < 0.01, statistically significant group difference

Figure 3 presents the results regarding prohibited substances in OTC medicines and dietary supplements (Table 1, Q8 and Q9). Over half of respondents (228, 59%) indicated that they knew OTC medicines and dietary supplements might contain prohibited substance (Table 1, Q8; Fig. 3a). For the PL and NPL groups, the numbers were 69 (85%) and 159 (52%), respectively (Fig. 3b), and this difference was significant (*p* < 0.01). Of those who answered yes to Q8, 29 (13%) reported knowing that the name of the prohibited substance might not be represented on the ingredient label of a dietary supplement (Table 1, Q9; Fig. 3c). There was no significant group difference on this question: 11 (16%) respondents were from the PL group and 18 (11%) were from the NPL group (Fig. 3d).

### The relationship between athletes and the medical professions regarding anti-doping activities

**Fig. 4:**
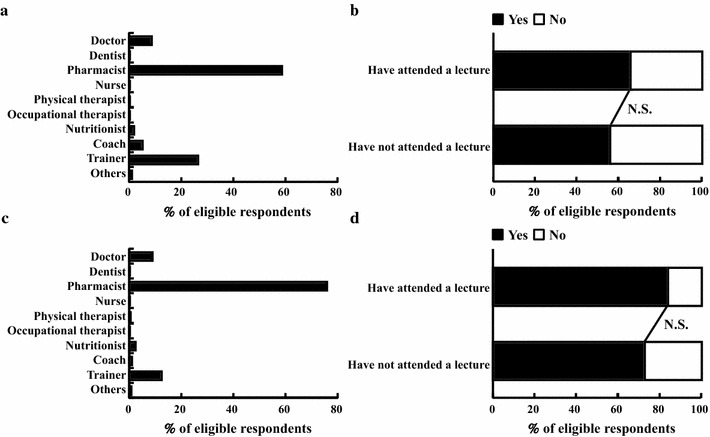
Results for the relationship between athletes and the medical professions. **a** Q10 (n = 374). **b** Q10 group comparison comparing those who had (n = 80) and had not (n = 294) attended a lecture about doping. **c** Q11 (n = 377). **d** Q11 group comparison comparing those who had (n = 80) and had not (n = 297) attended a lecture about doping

Results regarding the relationship between athletes and the medical professions (Table 1, Q10 and Q11) are shown in Fig. 4. A slight majority of respondents (218, 58%) thought that pharmacists should play a primary role in anti-doping activities for athletes (Table 1, Q10; Fig. 4a). For the PL group, the number was 53 (66%), and for the NPL group it was 165 (56%) (Fig. 4b); the group difference was non-significant. Three-quarters of respondents (285, 76%) thought that pharmacists’ role should be mainly as an advisor about dietary supplements (Table 1, Q11; Fig. 4c). In the PL group, 67 (84%) endorsed this statement, whereas in the NPL group, 218 (73%) endorsed it (Fig. 4d); however, this difference was also non-significant.

### Willingness to attend a future lecture about doping

**Fig. 5:**
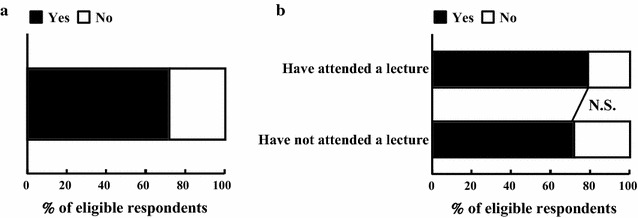
Willingness to attend a lecture about doping in the future. **a** Q12 (n = 406). **b** Q12 group comparison comparing those who had (n = 81) and had not (n = 305) attended a lecture about doping

Results regarding willingness for further education about doping (Table 1, Q12) are shown in Fig. 5. A large majority of respondents (291, 72%) indicated a desire to attend a future lecture about doping (Fig. 5a). The number in the PL group was 64 (79%) and in the NPL group was 219 (72%) (Fig. 5b); this difference was not significant.

## Discussion

Previous study in Japan indicates that it is important that people have the opportunity to consult with pharmacists in community pharmacy, as this will improve awareness of health and appropriate use of medication [14]. In addition, pharmacists have a potentially significant role to play in doping prevention and in promoting the rational, safe, and effective use of medications in sport [15–17]. We have reported the levels of pharmacy students’ recognition of various aspects of doping and their desire for education on this topic. Almost all respondents (95%) at least knew the word doping (Fig. 1a). Because there are many opportunities to hear this word in everyday life, such as on TV news, we suspected that most students would be familiar with it. Although only a small percentage of respondents (21%) had attended a lecture about doping (Fig. 1b), a much larger percentage (72%) wanted to attend such a lecture in the near future (Fig. 5a). We infer from this that there is a demand among students to have increased opportunities to learn about doping for the future as they prepare to play an active role as sports pharmacists. It also supports the recommendations of WADA to increase education and awareness of anti-doping throughout the medical profession [1]. In addition, further investigation in other countries about pharmacy students’ seeking more information about doping in sports will be needed to discuss Japanese students’ seeking is higher or average compared with the students elsewhere. Previous study in France showed the majority (83%) of pharmacists (mainly females) consider that doping is a public health problem. They agree (88%) that pharmacists have a part to play in doping prevention but also agree (58%; mainly females) that they are poorly or very poorly informed to that effect [18].

False knowledge was reported by some respondents: 57 (15%) thought that athletes were permitted to use prohibited substances to improve their performance (Fig. 1c), and 110 (28%) thought that athletes were never permitted to use such substances, even for medical treatment (Fig. 1e). Both of these erroneous beliefs on the part of practicing pharmacists could be detrimental to athletes by interfering with their ability to take medicines appropriately. Actual or potential health risks to athletes of using prohibited substances is one of the criteria for doping violations; yet at least 15% of respondents demonstrated imperfect knowledge of this criterion by endorsing the view that athletes are permitted to use prohibited substances to improve their performance. As future pharmacists who will play a professional a role in establishing proper usage of medicines, students need to be aware of this information. Athletes may coincidentally suffer from a proven medical condition for which a prohibited substance is required. Under such circumstances, an important procedure, the therapeutic use exemption (TUE), is embedded within the WADA provisions, whereby certification authorizes time- limited use of a prohibited substance based on legitimate medical need and after validation by independent expert scrutiny. The requirements to satisfy a TUE are that (1) withholding treatment would cause significant harm to health, (2) the use of the prohibited substance will confer no ergogenic benefit beyond the return to normal health, (3) no non-banned alternative treatment exists, and (4) the therapeutic need is not due to previous use of any prohibited substance without a TUE [19]. Because this procedure is sometimes cumbersome for athletes, medical professionals—especially pharmacists—should assist in the application process. To achieve this in the future, pharmacy students should have opportunities during their education to learn how to apply for a TUE, because they currently lack such knowledge.

It is important for pharmacy students to learn about past doping cases so that they can take advantage of such knowledge to support athletes more appropriately. Most anti-doping violations are committed when athletes unintentionally use prohibited substances [6, 7]; however, only 48% of respondents knew that this was the case in Japan (Fig. 2a), and only 34% knew about particular past cases of doping (Fig. 2c). Cases of which they were aware tended to be instances of intentional doping or doping at international competitions (Fig. 2d, e). We suspect that these results can be explained by the focus that such cases have received in popular media like TV. Therefore, pharmacy students need to learn from additional sources about unintentional doping and doping at domestic competitions, which is an issue to which they have a greater chance of being exposed.

Most respondents (59%) knew that OTC medicines and dietary supplements might contain prohibited substances (Fig. 3a). It has been reported that the name of a prohibited substance might not be represented on the supplement’s ingredient label [20], but only 13% of our respondents were aware of this (Fig. 3c). Those who think that OTC medicines and dietary supplements do not contain prohibited substances might support their use by athletes without checking their ingredients. If pharmacists do not know that some ingredients could be absent from the ingredient label, they might recommend a supplement without hesitation, even after checking the label. Athletes can be placed at a disadvantage by the pharmacist’s lack of such knowledge; therefore, students need to acquire this knowledge as part of their education.

Over half (58%) of respondents felt that pharmacists’ anti-doping involvement should be primarily in anti-doping activities for athletes (Fig. 4a); however, 76% thought the pharmacist’s role should be mainly as an advisor on dietary supplements (Fig. 4c). Pharmacy students are aware of their standing as future pharmacists who will play a professional role in society’s appropriate use of medicines. In addition, because 72% of respondents said they would like to attend a lecture about doping in the near future (Fig. 5a), if lectures or other education opportunities become more available, this will likely boost interest further, in addition to providing appropriate knowledge.

Our findings suggest that attending a lecture on doping produces some changes in knowledge. Specifically, knowledge that most doping violations in Japan are unintentional and that OTC medicines and dietary supplements might contain prohibited substances was significantly greater for those who had attended a lecture. In addition, attending a lecture appeared to influence the kinds of past cases of doping that pharmacy students recognized: knowledge of unintentional doping and of doping at domestic competitions was higher in the PL group (Fig. 2f, g). These results suggest that attending a lecture about doping may allow pharmacy students to be less affected by popular media like TV, which tends to focus more on intentional doping or doping at international competitions that draw high public interest. This difference could have a large influence on anti-doping activity in Japan in the future. In contrast, some areas of knowledge seem to have been unaffected by having attended a lecture (Figs. 1d, f, 3d, 4b, d). These areas also play a very important role in sports pharmacists’ support of athletes. Therefore, when Universities prepare their curriculum to address doping in the future, content relevant to these areas should be included and creatively developed to ensure high interest among pharmacy students. In addition, it is important for sports pharmacists to have a relationship not only with athletes and coaches but also with other pharmacists and pharmacy students, because there will also be a need in the future for general pharmacists to have appropriate knowledge of doping and supplement use. Lectures for the general public are also valuable, because appropriate knowledge of anti-doping activities will contribute to the formation of public opinion and will help demonstrate the significance of the pharmacist’s role. The findings and suggestions offered in this study can help us and others prepare new lectures that incorporate the needed content; this new curriculum should be evaluated with more advanced research techniques in the near future. For an international audience, especially people related to pharmacy education, we would like to ask to include the education of doping in the curriculum of pharmacy school for all students because all pharmacists might have opportunity to advise taking medicine to athlete at general community pharmacy.

Our study has several limitations. First, almost half of respondents’ data were not usable. About this, we believe they withdrew consent and left the room based on their rights because we told that they could indicate their consent by participating in this study. Second, although it was not a small study, it was conducted at single institution, Showa University. Third, in the present study, because we just asked them that “Have you ever attended a lecture about doping before?” we do not know exactly the length and contents of the doping lectures that students had attended. They were not specified and may have differed considerably. Nevertheless, our study offers new insights about pharmacy students’ knowledge of doping, the effects of a doping lecture on their knowledge, and their willingness to attend such a lecture in the future.

## Conclusions

We investigated pharmacy students’ recognition of certain facts about doping and explored the need for education in this area in Japan. Our results raise some awareness of areas of educational need. We also present evidence that pharmacy students already have enough interest and that attending a lecture would be useful for them. More education on doping for pharmacy students would be effective for anti-doping activities and would also benefit athletes.

## Abbreviations

WADC: World Anti-Doping Code
WADA: World Anti-Doping Agency
OTC: over-the-counter
TUE: therapeutic use exemption
